# Fracture of the Base of the First Metacarpal Bone

**Published:** 2013-01-18

**Authors:** Heather Kong, Janet H. Yueh, Mark Granick

**Affiliations:** ^a^Department of Orthopedic Surgery; ^b^Division of Plastic Surgery, University of Medicine and Dentistry of New Jersey, Newark

**Figure:**
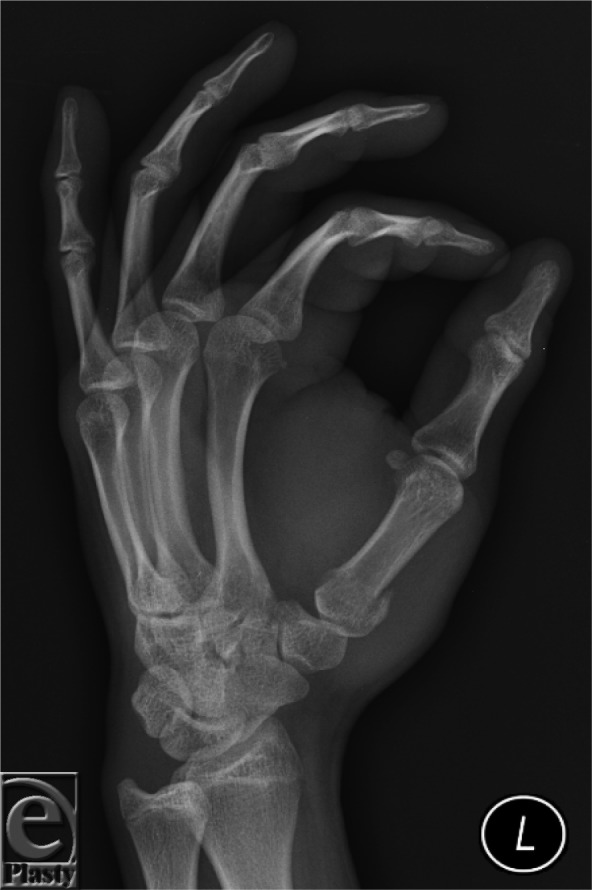


## DESCRIPTION

A 13-year-old baseball catcher presents with left thumb pain after a collision with another player.

## QUESTIONS

1. **What is the eponym for this fracture?**
2. **Describe the anatomy of the typical displacement of fracture fragments.**
3. **What are the indications for operative treatment of this fracture?**

## DISCUSSION

Fractures of the base of the thumb metacarpal can be divided into intra- and extra-articular fractures. The intra-articular fractures can be further subdivided into Bennett and Rolando fractures. The radiograph shown earlier illustrates a Bennett fracture. This fracture was initially described by Bennett in 1882.^1^

The injury occurs when the thumb metacarpal is axially loaded and partially flexed. The fracture line has a typical oblique course creating a small triangular fragment on the volar-ulnar aspect of the metacarpal base. This fragment is tethered by the uninjured anterior oblique ligament, while the main portion of the thumb metacarpal displaces radially, proximally, and dorsally by the pull of the abductor pollicis longus and the adductor pollicis.

Whether a Bennett fracture should be treated by open or closed reduction is still a subject of discussion. Closed reduction of a Bennett fracture can be attempted by applying a wet plaster thumb spica dressing. As the plaster dries, traction is placed on the thumb to pull the metacarpal distally with concurrent pressure pushing the metacarpal base ulnarly and volarly to return it to anatomical position. If anatomic reduction is obtained, a nonoperative course may be pursued. However, close follow-up with frequent radiographs is necessary as redislocation and fracture redisplacement are common after conservative treatment. Long-term results have shown that persistent subluxation of the first carpometacarpal joint can result in marked degenerative changes and poor function.^2^

Most authors now recommend improvement of the metacarpal subluxation with percutaneous or open reduction. Operative treatment with closed reduction and percutaneous pinning is often attempted first. Percutaneous Kirschner wires can be inserted through the base of the metacarpal, across the joint, and into the trapezium to hold the reduction in place. This wire remains in place for a period of 4 weeks, at which time the wire is removed and a rehabilitation program is started. If one cannot achieve anatomical closed reduction with an intra-articular step off of not more than 2 mm, then open reduction and internal fixation should be performed.^3^ Under direct vision, either Kirschner wires or lag screws can be inserted to hold the reduction in place. Postoperatively, if pins are used, the thumb is immobilized in a thumb spica cast for 4 weeks. Because screw fixation is more secure, active range of motion may be initiated 5 to 10 days postoperatively. Long-term functional outcome studies have shown that strength of the affected hand is decreased in all patients regardless of the type of treatment.^4^ While most patients have some degenerative changes radiographically, surprisingly there is no correlation with symptoms.
